# Middle-Eastern plant communities tolerate 9 years of drought in a multi-site climate manipulation experiment

**DOI:** 10.1038/ncomms6102

**Published:** 2014-10-06

**Authors:** Katja Tielbörger, Mark C. Bilton, Johannes Metz, Jaime Kigel, Claus Holzapfel, Edwin Lebrija-Trejos, Irit Konsens, Hadas A. Parag, Marcelo Sternberg

**Affiliations:** 1 Department of Evolution and Ecology, University of Tübingen, Auf der Morgenstelle 5, 72076 Tübingen, Germany; 2 Faculty of Agriculture, Food and Environment, Hebrew University of Jerusalem, Rehovot 76100, Israel; 3 Department of Biological Sciences, Rutgers University, 195 University Avenue, Newark, New Jersey 07102, USA; 4 Department of Molecular Biology and Ecology of Plants, Tel Aviv University, Tel Aviv, 69978 Israel

## Abstract

For evaluating climate change impacts on biodiversity, extensive experiments are urgently needed to complement popular non-mechanistic models which map future ecosystem properties onto their current climatic niche. Here, we experimentally test the main prediction of these models by means of a novel multi-site approach. We implement rainfall manipulations—irrigation and drought—to dryland plant communities situated along a steep climatic gradient in a global biodiversity hotspot containing many wild progenitors of crops. Despite the large extent of our study, spanning nine plant generations and many species, very few differences between treatments were observed in the vegetation response variables: biomass, species composition, species richness and density. The lack of a clear drought effect challenges studies classifying dryland ecosystems as most vulnerable to global change. We attribute this resistance to the tremendous temporal and spatial heterogeneity under which the plants have evolved, concluding that this should be accounted for when predicting future biodiversity change.

## Abstract (web summary)

Semi-arid and Mediterranean ecosystems are predicted to be vulnerable to climate change. Here, Tielbörger *et al*. show that plants along a steep climatic gradient in a biodiversity hotspot are resistant to both irrigation and drought in multiple years of experimental rainfall manipulation.

## Graphical abstract

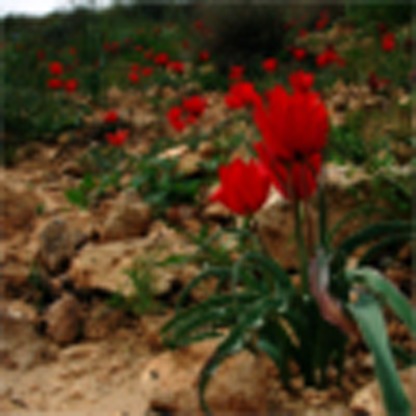

The Eastern Mediterranean Basin is a global biodiversity hotspot^1^, which has been under human influence for millennia, and harbours valuable genetic resources for some of the world’s most important crop species^2^. It is also one of the few regions for which climate models agree in their predictions: decrease in precipitation, increase in temperature and increase in temporal climatic variation^3^. Because semi-arid and Mediterranean ecosystems are highly water-limited and under heavy human pressure, numerous theoretical studies have classified them among the most vulnerable to global change^4^,^5^,^6^.

Unfortunately, empirical evidence supporting these predictions is scarce. Long-term experiments (>5 years) manipulating precipitation in drylands are extremely rare^7^,^8^,^9^, they seldom^10^,^11^,^12^ include more than a single site^13^ and usually examine perennial plants which are less likely to respond within a short time period^10^,^11^,^12^,^14^,^15^,^16^. Also, they often focus on functional, physiological and ecosystem-level responses rather than variables of community structure^7^, which are more relevant for assessing species extinction risk and biodiversity-driven ecosystem services.

Due to this lack of long-term experimental information, predictions of future species diversity and distributions are commonly based on bioclimatic envelope models (BEMs^17^), which deduce climatic requirements for individual species from their current geographical range and assume migration of species in parallel with the shifting climate to which they are adapted^6^,^16^,^17^,^18^,^19^,^20^,^21^. Such range shifts were also classified as ‘likely’ across a wide range of biomes in the latest IPCC (Working Group II) report that summarizes the observed and potential impact of climate change on biodiversity^22^. However, despite their attractiveness, limitations of BEMs have been widely reported, for example, unrealistic dispersability, simplified niche structure, disregard of biotic interactions^23^ and of plastic or adaptive responses of plants^24^ to climate change^7^,^17^,^25^,^26^,^27^. To truly assess the vulnerability of ecological communities and validate these correlative large-scale models, it is desirable to assess their predictive power with field experiments founded on ecological mechanisms^17^. This is a large challenge because even some of the leading researchers in BEM modelling have argued that model validation could be conceptually impossible^28^ as it can only be done retrospectively and by correlative inference, for example, after species migration has been observed^21^,^29^.

Here, we present results from long-term climate manipulations that combined the convenient logic of BEMs with experimental testing of their predictions under field conditions. BEMs commonly assume that community shifts are due to migration of species to catch up with their climatic niche, but frequently rely on unrealistically high dispersal abilities of plants^29^,^30^. However, the same prediction for changing community structure also applies to a more realistic *in situ* response of the resident species^17^,^24^, which may lead to an increase or decrease in local abundance. Namely, decreasing precipitation will lead to an *in situ* increase in dry adapted species at the expense of wet adapted ones and vice versa, due to preferential selection of potentially pre-adapted species from an existing species pool. Our innovative experiments were designed to test whether such a directional shift in species composition will occur when plant communities are exposed to many years of climate manipulation in the field. The approach is based on the assumption that under climate change, structure and function of resident plant communities follow a trajectory away from the current status and towards those plant communities that inhabit such climatic conditions today (Fig. 1b). This assumption can in fact be tested experimentally if the investigations are conducted along a climatic gradient.

An ideal system for this novel type of experiment should exhibit high species richness, an overlapping species pool across a large range of climatic conditions, and include short-lived organisms providing the potential to respond within a relatively short time. Unfortunately, though multi-site studies have been advocated, especially for studies of species compositional change^13^,^31^, such conditions have rarely been fulfilled. Here, we studied the impact of rainfall manipulations on highly diverse annual plant communities across a steep rainfall gradient in Israel (Fig. 1) for 10 consecutive growth seasons, comprising one baseline year and nine manipulated years. Annuals were studied as they account for most of the species and annual net primary production—ANPP^32^,^33^, that is, biomass of annuals is a suitable proxy for overall site NPP. Four sites sharing many environmental characteristics but exhibiting an 8-fold difference in annual rainfall between driest and wettest sites were located in arid (A), semi-arid (SA), Mediterranean (M) and mesic-Mediterranean (MM) climates (Fig. 1a). Rainfall was experimentally decreased or increased by 30% in SA and M sites, using rainout shelters^34^ and irrigation systems, respectively. The dry treatment mimicked recent regional climate change scenarios^3^,^35^. We monitored community response (biomass, density, diversity, species composition) annually in two microhabitats (open areas versus areas under shrubs). We felt this was essential, because shrubs can act both negatively (competition) and positively (facilitation) on many annual species depending on their location^36^, causing differences in the potential climatic responses between open patches and shrub understory. The innovation of our multi-site approach lies in the fact that community responses to climate manipulations were compared to two contemporary controls, *in situ* control plots and control sites with rainfall similar to the manipulations. This enabled us to both test for predictions of correlative models based on geographic gradients as well as generating mechanistic information for community response to rainfall within sites (Fig. 1b). Our overall findings suggest that correlative models are a poor proxy for predicting plant community response to climate change. Where these models predict a strong response of plant community structural variables to a changing climate, the results from the long-term and multi-site experiments indicate a very high resistance of the studied plant communities to manipulated rainfall. This resistance is likely due to the fact that climate change could be well within the range of the large climatic variation that is characteristic for the studied ecosystems.

## Results

### Site conditions and abiotic variables

Rainfall monitoring during the study period confirmed the steep aridity gradient and indicated that mean annual precipitation decreased and rainfall variation increased towards the arid site (Fig. 2). Soil measurements confirmed that the applied rainfall manipulations (Supplementary Fig. 1) had significant effects on soil moisture, especially in the drought treatment. Soil moisture was significantly lower in the drought plots (~28% average reduction), intermediate in control plots and highest (~8% increase) in irrigated plots (Fig. 3, Supplementary Table 1). There was a tendency for the irrigation to have a stronger effect in the Mediterranean site. Temperatures were also increased in drought plots (average increase 0.6 °C, no effect under shrubs in SA) and decreased in irrigated plots (average decrease 0.9 °C, with smaller effect in M), further adding to the realism of our manipulations with respect to mimicking climate change.

### Vegetation patterns along the climate gradient

**Figure 1:**
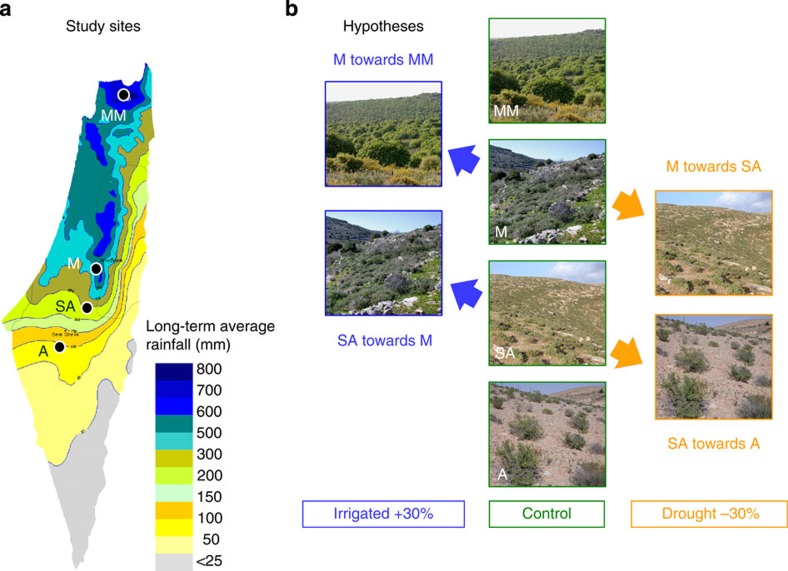
Illustration of rationale and hypothesis of the study. (**a**) Map of the study region with isohyets (average annual rainfall) and locations of four research sites in arid (A), semi-arid (SA), Mediterranean (M) and mesic-Mediterranean (MM) climatic regions. Long-term annual average in the four sites is 90 mm (A), 300 mm (SA), 540 mm (M) and 780 mm (MM), respectively. (**b**) Visualization of hypothesis for the four research sites along the climate gradient with two contemporary controls. *In situ* control plots determined whether the treatments were having any effect on community structure, controls in neighbouring sites were used for testing for the direction of change. Namely, correlative models predict that community parameters under manipulated rainfall (irrigation, drought) should become gradually more similar to those in corresponding adjacent climates (that is, drought treatment: semi-arid towards arid; Mediterranean towards semi-arid; irrigated treatment: Mediterranean towards Mesic-Mediterranean; semi-arid towards Mediterranean). As species pools are overlapping among sites and dispersal is highly limited^38^, we assumed gradually increasing convergence of adjacent sites to occur via *in situ* changes of communities. Note that the dry treatment corresponds to recent climate scenarios^3^,^35^, but both treatments yield mechanistic insight into vegetation–rainfall relationships.

The univariate response variables in control plots showed large and often significant differences among the four sites along the natural climatic gradient (Fig. 4). This suggested that there was the capacity for a potentially strong response to the *in situ* climate manipulations, too. These findings provided the prediction for the direction of change in the manipulated plots according to the rationale explained in Fig. 1. The predictions for the change in community structure in the drought treatment in a given site were given by the community variables in the adjacent drier site. These were unequivocal and suggested that drought, which simulated climate change, should lead to a decrease in mean values of biomass, species richness and density in both sites and both microhabitats (Fig. 4). The predictions for the irrigation treatment in the semi-arid site were also consistent and suggested an increase in mean values of these three response variables. However, the predicted direction of change in the wet treatment of the Mediterranean site, given by the comparison with the MM site, were less straightforward. Species richness and density under shrubs were lower in the MM than in the M site suggesting that experimental irrigation would decrease mean values for richness and shrub understory density in the M site (Fig. 4). Density in open areas and biomass under shrubs would be predicted to stay the same under irrigation, while biomass in the open areas should increase with increased water supply.

### Vegetation response to rainfall manipulation

**Figure 2:**
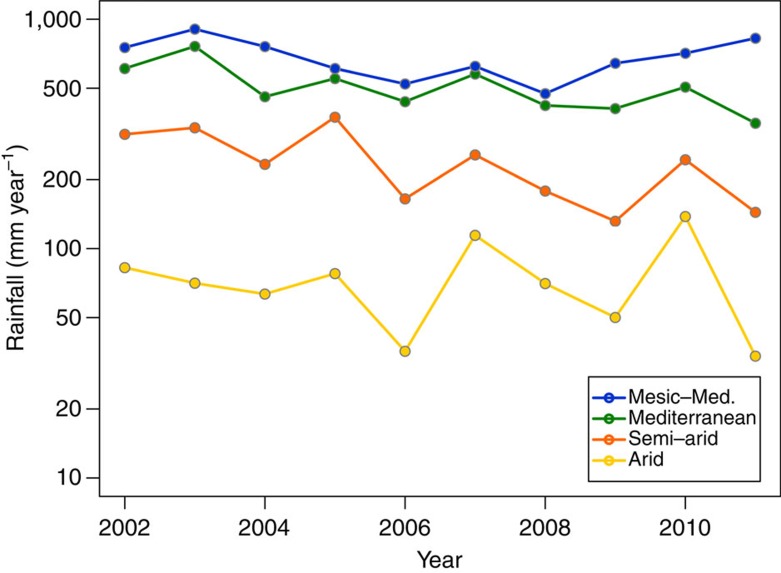
Total annual rainfall during the 10 consecutive study years. Inter-annual variation in rainfall increases from the mesic climates to the arid ones. There was a trend of decreasing annual rainfall during the study period in the three wetter sites. Note that the scale was log transformed to better visualize the larger variation in annual rainfall towards the drier sites.

In general, all response variables varied greatly and significantly among years (Table 1) and these annual differences in plant density, diversity, biomass and species composition were much larger than the effects caused by the rainfall treatments (Fig. 5, Supplementary Fig. 2). Interestingly, these annual variations were not unequivocally correlated with rainfall differences among years (Fig. 2). Despite our predictions, and the clear difference in abiotic conditions between wet and dry plots, there was no difference between these two extreme treatments in any response variable. Multivariate analyses showed no rainfall manipulation effect on species composition at either site, indicated by the lack of significant year × treatment interactions (Table 1). Despite species composition varying greatly among years, trajectories for temporal changes in community structure were largely parallel in the three treatments (Fig. 6, Supplementary Fig. 2). Consequently, plant communities experiencing 9 years of drought did neither diverge from controls within a site nor did they gradually approach *in situ* the structure of communities in adjacent drier stations. Likewise, irrigated plots did not become more similar to the structure of the corresponding wetter site, nor differ from *in situ* controls.

Data for the univariate parameters biomass, species richness and density could be described better when including information from 1 and 2 years before the growth season (see Methods, Supplementary Methods) rather than incorporating just growth season alone. Even so, all of these parameters showed very little response to the rainfall manipulations (Fig. 5). Only a few weak or inconsistent patterns were detected when looking into separate variables (36 pairwise treatment comparisons altogether, none significant when correcting for multiple testing), and there were no differences between wet and dry plots. Throughout the experiment, there were clear responses of above-ground annual biomass to natural rainfall levels. Biomass increased towards wetter sites (Fig. 4), it varied greatly among years, and was consistently lower under shrubs relative to open areas (Fig. 4, Fig. 5). However, compared with these natural spatio-temporal variations, differences in biomass among treatments were small and only communities under shrubs of the drier SA site responded positively to irrigation over time (irrigation > control, Fig. 5b). Species richness varied among sites and years but generally not among treatments (Fig. 5). Surprisingly, overall species richness was found to be significantly higher in the drought treatment than in controls in open patches at the SA site, but this difference did not increase with time. For density, again site and year effects were significant. However, only a single treatment effect was observed in the open patches at the wetter Mediterranean site (drought < control, Fig. 5k).

## Discussion

Overall, the plant communities responded very little to 9 years of rainfall manipulation. There was no response of overall species composition, and only three significant changes were observed in 36 pairwise comparisons of community structure—a visibly increased biomass under irrigation, a subtle increase in species richness and a decrease in density under drought. Because downscaled scenarios suggest increasing aridification in the study region within the range of our drought treatment^3^,^35^, and because the drought treatment had a larger effect on water availability, we conclude that our Mediterranean and semi-arid annual plant communities would be little affected by climate change, at least in the short-to-medium term. For communities previously classified as particularly vulnerable to change^4^,^5^,^6^,^18^, these findings are intriguing, especially as the long-term nature of the manipulations, across multiple sites, with many species and subjected upon nine generations of annual species, was ideal for eliciting a community response.

Similarly small or delayed responses to long-term rainfall manipulation on plant community structure, albeit in herbaceous perennials, have been detected in water-limited temperate grasslands^10^,^14^,^16^,^37^. These were attributed to fine-scale heterogeneity in environmental conditions^16^, allowing for intra-specific genotype selection instead of changes in mean abundance of species. This explanation may also apply to our sites, where spatial variation is considerable, occurring at a centimetre scale^38^. However, a more parsimonious explanation for the apparent resistance to change seems to be past selection for species and genotypes exhibiting specific adaptations^39^ for coping with the large natural temporal variation in rainfall. This variation is typical for all arid to Mediterranean ecosystems. Climatic extremes with extended drought periods have characterized our study region for millennia^40^,^41^, and may have inspired biblical accounts such as of the legendary 7 years of famine. To date, inter-annual variation in rainfall in the Middle East commonly exceeds the magnitude of the change predicted by global and regional climate scenarios^3^,^35^,^42^ which were mimicked by our drought treatment.

A combination of adaptations to climatic variation in subtropical drylands may dampen species response to climate change. For example, high drought resistance and phenotypic plasticity^43^ enable reproduction even under limited rainfall. Moreover, bet-hedging mechanisms, in particular, seed dormancy^44^,^45^, buffer catastrophic years and detrimental effects of environmental variation on population growth. Persistent seed banks may therefore also have buffered the response to the rainfall treatments, especially of highly dormant species such as legumes. An alternative risk-spreading mechanism is large seed size, which ensures high survival rates despite strong environmental variation^46^. Large-seeded species are abundant in our study system^46^ and several of those, such as wild barley, oat or chickpeas are progenitors of important modern crop species. Though longer studies are always desirable, the observed lack of response of the herbaceous community to nearly a decade of rainfall manipulation seems robust and likely resulted from various adaptations to the naturally large variation in rainfall through buffering mechanisms. Such mechanisms could also be the basis of so-called legacy effects^47^, positive correlations between ANPP in consecutive years that have been identified as a reason for delayed ANPP response to climate change in perennial plants. In annual plant communities such as ours, similar legacy effects could be particularly common because high performance in a given year should have direct consequences for densities in the following year.

Despite the evidence for community resistance, some subtle changes were detected within our experiment. However, caution should be applied when interpreting these few significant results out of 36 pairwise comparisons, especially when no differences between wet and dry treatments were observed.

Prompt responses of biomass to climate manipulation could be expected, as it is so tightly linked to rainfall in a given year. Indeed, the only pattern detected was a subtle increase in biomass, albeit only for irrigated shrub understory communities at the drier site. This site-specific pattern matches large-scale patterns found in this study and across the region^32^, where herbaceous ANPP responded to precipitation variation only in sites with 300 mm annual rainfall or less. A cumulative effect of consistently changing conditions on ANPP could be responsible for these site-specific responses, because such legacies are more common in dry than in wetter systems^47^. The fact that only irrigation, but not drought, caused a biomass response is surprising given that the drought treatment had a much stronger effect on soil moisture. However, such a pattern has also been found previously^48^ and corroborates our conclusion of a general resistance of our systems to climate change. Support for our findings was also obtained in a recent empirical and modelling study conducted in semi-arid and Mediterranean systems in the same region^33^. Here, the observed high resilience in annual plant biomass production was found to break down only when taking into account much more drastic decreases in precipitation than those currently assumed.

The counterintuitive increase in species richness with drought observed in the open areas of the semi-arid site lacked a significant trajectory and was not corroborated by differences between dry and wet plots. It thus seems likely that this merely reflects differences among plots at the outset of the study. The same could apply for the second response to the drought treatment, a subtle decrease in plant density in open patches at the wetter M site, because it did not differ from the irrigation. Alternatively, densities in the Mediterranean community could be more responsive because the adaptations that potentially buffer plant abundance change to altering climate are less prominent than at the drier SA site^43^,^44^. Seed dormancy, for example, could be responsible for a delayed response, and indeed these patterns were not present until after 8 years of manipulation.

Multi-site climate change experiments have recently been strongly advocated^13^,^31^ but our experiment is unique in combining two common approaches for climate studies: *in situ* manipulations which are mechanistic and mimic temporal community shifts; and strict environmental gradients founded on space-for-time assumptions. Furthermore, to the best of our knowledge, there is also no previous study spanning across nine plant generations, because most long-term studies have been conducted with perennial plants (Supplementary Table 5). Comparing our findings with other studies must therefore be done with caution. The few longer-term (≥5 year) drought experiments looking at community structure and performed in single sites in semi-arid US and Spain, showed large inter-annual variation and a delayed negative effect on primary productivity of perennial vegetation^49^, an erratic response of short-lived ruderal species^8^,^49^ or other species-specific responses^12^. In Mediterranean ecosystems in California, no response in annual plant species richness was observed to moderate additional winter irrigation^23^ and some increase in herbaceous biomass under much more extreme rainfall manipulation^50^. A small-scale study in Spain, in a site where annuals were rare, showed species-specific responses of shrubs to experimental drought^15^ but these were attributed to the highly dynamic successional nature of these post-fire communities.

Common to almost all longer-term experimental studies focusing on community structure and biodiversity in drylands is that they suggest some resistance to manipulated climate. In part this may be related to lack of experimental power (due to limited replication or spatial and temporal extent) or a delayed change in species composition due to buffering mechanisms^44^,^45^,^47^. However, the growing evidence does seem to suggest that in systems with large natural climatic variation, such as arid to Mediterranean ecosystems, the community responses are less pronounced than those predicted purely by correlative models^8^,^33^,^39^. This indicates that these systems may be less vulnerable to the effects of climate change than previously thought. The reason for this resistance most likely lies in the fact that the predicted changes in climatic conditions and accordingly, the magnitude of manipulative treatments, lies well within the ‘climatic comfort zone’ to which the component species are adapted. This can also be seen by comparing the magnitude of between-year variation with treatment effects: annual variations in all community variables were two to three times larger than the largest differences among treatments in single years. However, even in our comprehensive study, we cannot rule out that species composition may change after a longer lag phase^13^,^47^, because natural short-term climatic variations may impose a different selection regime on organisms than a long-term trend of changing rainfall or temperature. We therefore advocate for long-term experiments, ideally along climatic gradients (for example, precipitation, elevation), which explicitly address the role that inherent adaptations to both the mean and the variability in current climate conditions may have in plant response to climate change. Accurate and reliable assessments of ecosystem vulnerability are extremely important for informed and cost-effective adaptive management under climate change, especially of highly diverse ecosystems such as ours which harbour important genetic resources^2^ and are at the same time under heavy human pressure^4^. Combining correlative models based on the magnitude of current and projected climatic variability in space and time, with studies on the adaptive potential of species^24^,^39^, will allow for such assessments of the vulnerability of organisms and communities to climate change.

## Methods

### Sites and setup

Experiments were conducted at four research sites situated along a steep natural rainfall gradient that runs from Northern to Southern Israel (Figs 1 and 2). The overall climate is Mediterranean with mild, wet winters and hot, dry summers. The particular sites represent four different types of rainfall-driven ecosystems: arid (A-90 mm rainfall per year), semi-arid (SA-300 mm), Mediterranean (M-540 mm) and mesic-Mediterranean (MM-780 mm). Within- and between-season rainfall variability is high and increases towards the desert (Fig. 2). All sites are located over the same calcareous bedrock on south-facing slopes at similar altitudes and experience similar mean annual temperatures that range from 17.7 to 19.1 °C. Growth season length is determined by rainfall distribution, commencing usually in October-November and ending in April-May, with shorter seasons in drier sites.

The habitats are shrublands with open patches among woody plants. The vegetation below and between shrubs is dominated by annual species (see detailed site description in Supplementary Methods). Annual plants in the region include 70–95% of the plant species and up to 100% of the herbaceous vegetation, which in turn accounts for up to 100% of the ANPP^32^,^33^, especially under drier climates and higher grazing pressure. More reasons to study this key component of the community were its potentially fast response to rainfall variation, very high species richness (>600) with many shared species among sites (Supplementary Table 5), as well as its importance as main forage for livestock and as progenitors of globally important crop plants (Supplementary Table 5). Though shrubs, due to their slow growth, contribute much less to ANPP than annuals, they comprise a very distinct microhabitat for annual plants^36^ which covers 5, 25, 40 and 70% of the area in the arid, semi-arid, Mediterranean and mesic-Mediterranean sites, respectively.

**Figure 3:**
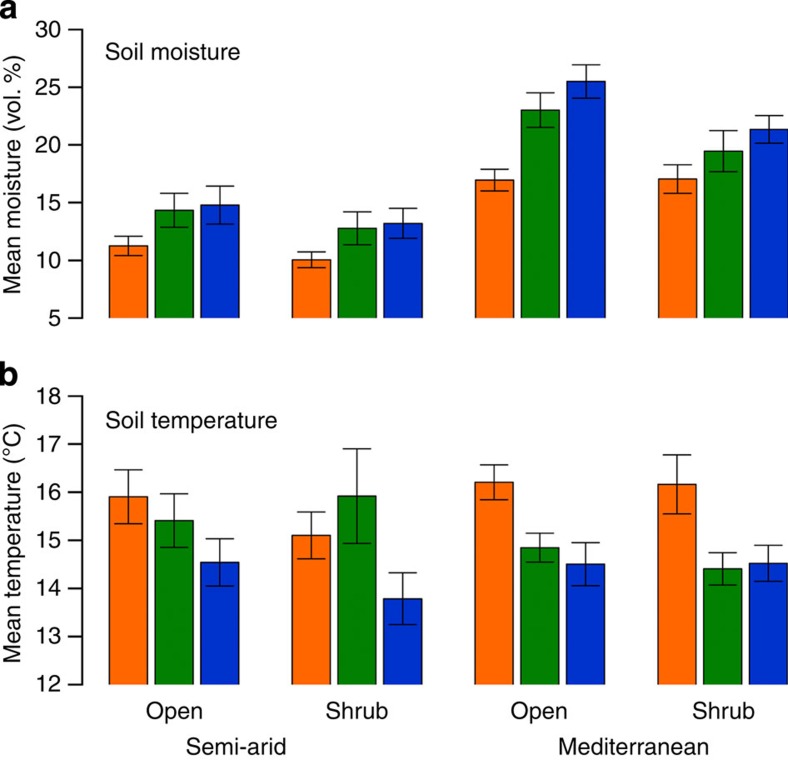
Average soil moisture and temperature in rainfall manipulation treatments. Means across years (±s.e.) are given during the first eight study years in two sites, two microhabitats (under shrubs, in open areas) and three rainfall manipulation treatments (dry—orange, control—green, wet—blue). Linear Mixed Models were used to compare the treatments. Treatment effects on soil moisture (**a**) were significant in all the cases and in the expected direction with soil moisture being significantly lower in the drought treatment, intermediate in control plots and highest in irrigated plots. Temperatures (**b**) were also increased in drought plots and slightly decreased in irrigated plots (except for plots under shrubs in the semi-arid), adding to the realism of the climate manipulations and confirming the robustness of the results.

**Figure 4:**
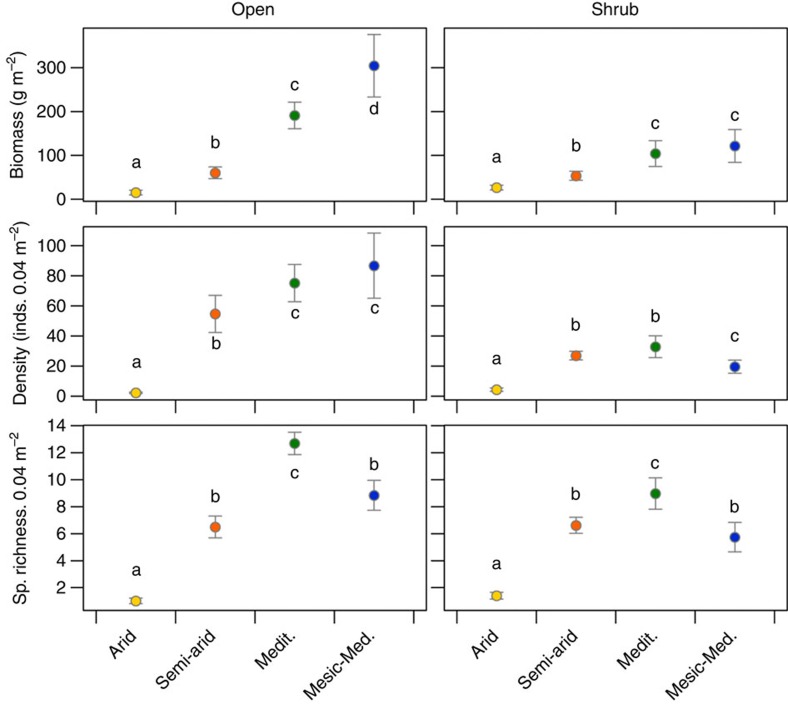
Mean above-ground biomass, plant density and species richness (sp. richness) under ambient (control) conditions. Data stems from open areas and areas under shrubs in four sites (indicated by four different colours) along the rainfall gradient, calculated across all 10 study years. These patterns provided the predictions for the response to the rainfall manipulations in the two intermediate sites, based on the space-for-time assumption of bioclimatic envelope models (yet assuming *in situ* change instead of migration of plants). Mean values with different letters are significantly different from each other within each panel (LMM Tukey–Kramer adjusted contrasts *P*<0.05; *N*=105 (biomass); *N*=120 (density and richness)). Note that although displayed on normal scale, biomass and density were statistically analysed on a log scale. inds., individuals.

**Figure 5:**
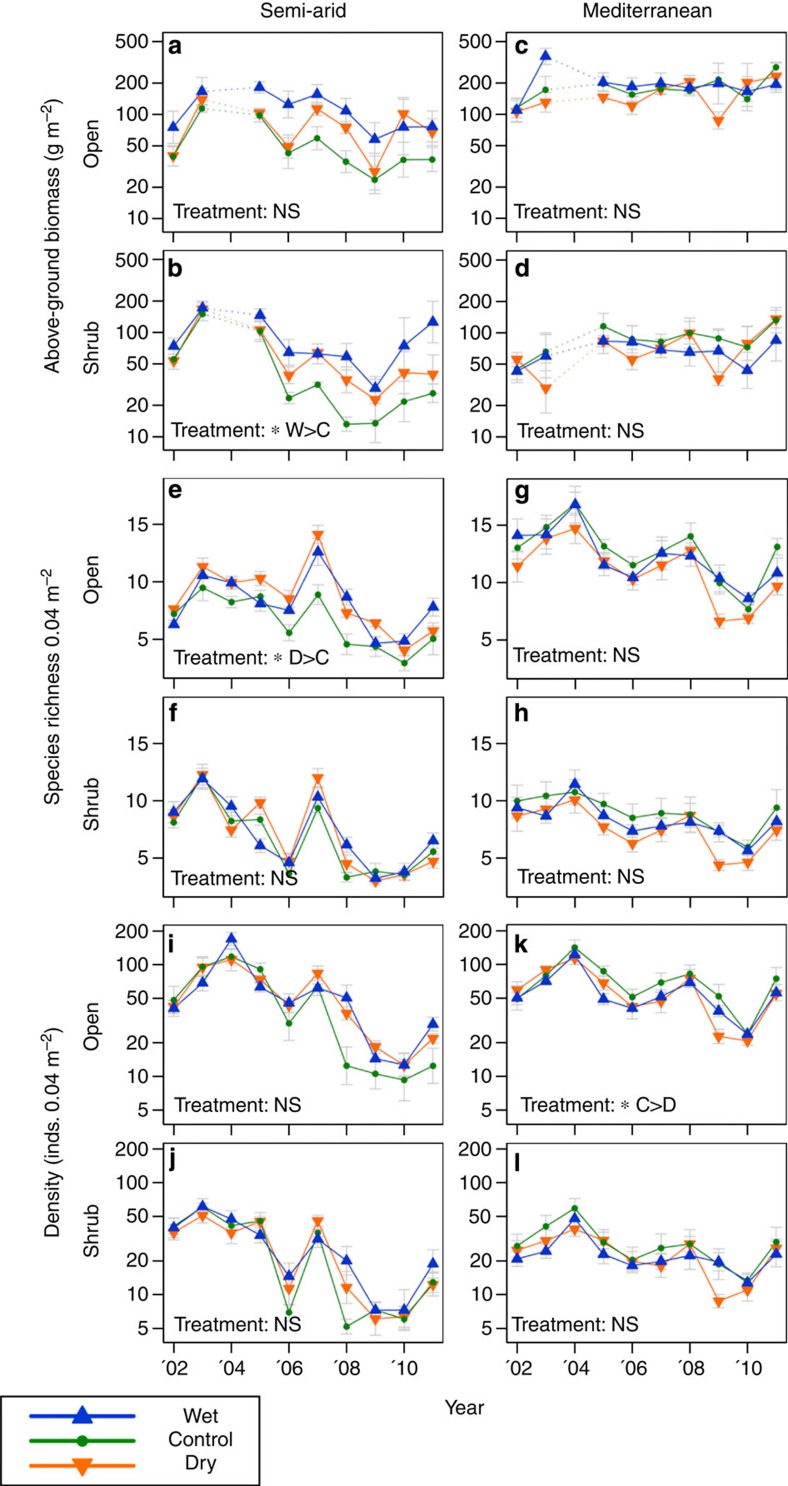
Response of three plant community variables to rainfall manipulation treatments. Above-ground biomass (**a**–**d**); species richness (**e**–**h**); and density (**i**–**l**). Data are expressed as mean (±s.e.). Above-ground biomass comprises of visual estimates calibrated by nearby destructive sampling, data for the 2003/04 season were missing. Density (number of individuals- inds.) and richness are expressed as mean per permanent quadrat (400 cm^2^). Linear Mixed Models were used to compare treatments (Supplementary Table 2), with overall treatment effects (DF=2,11; *N*=120 (biomass), or *N*=135 (density and richness)) indicated by an asterisk (*0.05>*P*>0.02) and significant contrasts (Tukey–Kramer adjusted) showing paired comparisons (w, wet; d, dry; c, control treatment). Of the 36 possible pairwise treatment comparisons, only 3 proved to have uncorrected *P* values <0.05, with no detectable difference between the wet and dry treatment in any variable: Biomass of under shrub communities at the semi-arid site showed a slight response to the wet treatment (wet > control *P*=0.028); species richness exhibited a minor treatment effect in open patches at the semi-arid site (dry > control *P*=0.031), and an opposite effect was detected for density of communities in the open patches of the Mediterranean site (dry < control *P*=0.021).

The sites were established in the 2001–2002 season which served as a baseline year for monitoring the vegetation before manipulation. Without such a reference year, real treatment effects cannot be distinguished from initial differences among plots. Rainfall at the M and SA site was then manipulated during nine consecutive growing seasons, 2002–2003 to 2010–2011. At each site, five plots of 10 m × 25 m were allocated as ‘control’ plots which received natural rainfall. At the two manipulated sites, 5 each of a further 10 plots were randomly assigned as either ‘dry’ or ‘wet’ treatments. Initial trials indicated that large retractable PVC rainout shelters, commonly used on much smaller scale manipulation plots, were not able to resist the strong winds that accompany major rainstorms. Therefore, natural rainfall was reduced in dry plots by v-shaped transparent plastic strips covering 30% of the area, as proposed previously^34^. The strips were installed onto a metal frame 2 m above ground level (Supplementary Fig. 1). They allowed passage of all photosynthetically relevant radiation, an even coverage of rainfall to reach the surface, and avoided unintentional side effects which are common with permanent shelters^34^ (Fig. 3, Supplementary Table 1). Wet plots were irrigated by drizzle sprinklers installed 1 m above the ground (Supplementary Fig. 1), and the rationale for application of rain was similar to most previous irrigation experiments^12^. The sprinklers added 10 mm precipitation to every other main rainfall event and were operated until precipitation was increased by 30% of the long-term annual rainfall at the sites—commonly in March (details in Supplementary Methods).

The magnitude of our treatments was inspired by low-resolution global circulation models with high uncertainty and a range including both increases and decreases in annual precipitation until 2099 (ref. 42). During the course of our study, ensembles of downscaled regional climate scenarios became available and suggested an increasing aridification in the region, that is, a decrease in annual precipitation, increasing temperatures and increasing climatic variability^3^,^35^. Our drought treatment, which also increased temperatures (Fig. 3) is similar to the more extreme predictions and is the relevant treatment for mimicking climate change response in our study systems. Nevertheless, the irrigation treatment adds to our understanding of community change in response to rainfall in general, it adds a second dimension to our aim of testing correlative models, and it allows for a possible greater effect *in situ* between treatments within a site.

### Data collection

Within each plot, 10 permanent quadrats (20 cm × 20 cm) were placed randomly in five pairs before the onset of rainfall in October 2001 and monitored until the end of the growing season in 2010/2011. To avoid edge effects, the quadrats were established at least 1 m away from the plot boundaries. One quadrat per pair was located under the canopy of the dominant shrub species, and the other one in open areas ~50 cm away from shrubs, to capture any important influence that shrubs may have on presence, abundance and performance of annuals in the system^36^. These influences can be very large and they are not the same in each site, with negative shrub effects on annual performance being more common in wetter sites^36^. Distinguishing these two microhabitats also addressed an important point of criticism related to BEM, which is the disregard of biotic interactions^23^. All permanent quadrats were monitored at peak vegetation season, that is, February–March at the arid site and March–April at the wetter sites. In each quadrat, plants were counted and identified. Some closely related species could not be unequivocally distinguished and were aggregated at the genus level. The final data set included 60, 103, 96 and 20 taxa in MM, M, SA and A, respectively (123 annual taxa altogether, Supplementary Table 5).

Above-ground biomass was assessed at peak season by nondestructive estimates of aerial cover multiplied by mean plant height in all permanent quadrats. These estimates were calibrated to absolute g m^−^^2^ values by linear correlation with dry mass of identical samples harvested outside our study plots (Supplementary Methods). The calibrations indicated that the visual estimates were a suitable proxy for above-ground biomass (*P*<0.001, M: *R*^2^=0.65; SA: *R*^2^=0.73; DF=31).

To test whether our treatments were having the intended effect on abiotic conditions, we recorded hourly soil moisture and temperature from 2002–2009. Four temperature (thermocouples) and four soil moisture (TDR) sensors per site and rainfall manipulation treatment and six sensors in controls were buried horizontally at 10 cm soil depth, split among the two major microhabitats—under shrubs and between shrubs.

### Data analysis

Community composition was analysed with multivariate constrained ordination (redundancy analysis) in Canoco 4.5 (ref. 51). Redundancy analysis is a commonly used technique to statistically test clear hypotheses for multivariate data sets, which in our case simultaneously takes into account any potential changes in the abundances of all single species in our communities with respect to the manipulations. Before the analyses, the species abundance data of the five subsamples per microhabitat and plot were pooled and square root transformed. Following the rationale of correlative models, we tested whether the temporal trajectory of community composition differed between treatments, that is, year × treatment, and in the hypothesized direction (Fig. 1b). This was done separately for each site and microhabitat using year as continuous predictor and plot identity and single years as covariates to control for potential differences between plots before the onset of treatments. A second test assessed the overall variation among single years (here: categorical). Significance was assessed with Monte Carlo tests with 999 permutations adjusted to the model structure.

**Figure 6:**
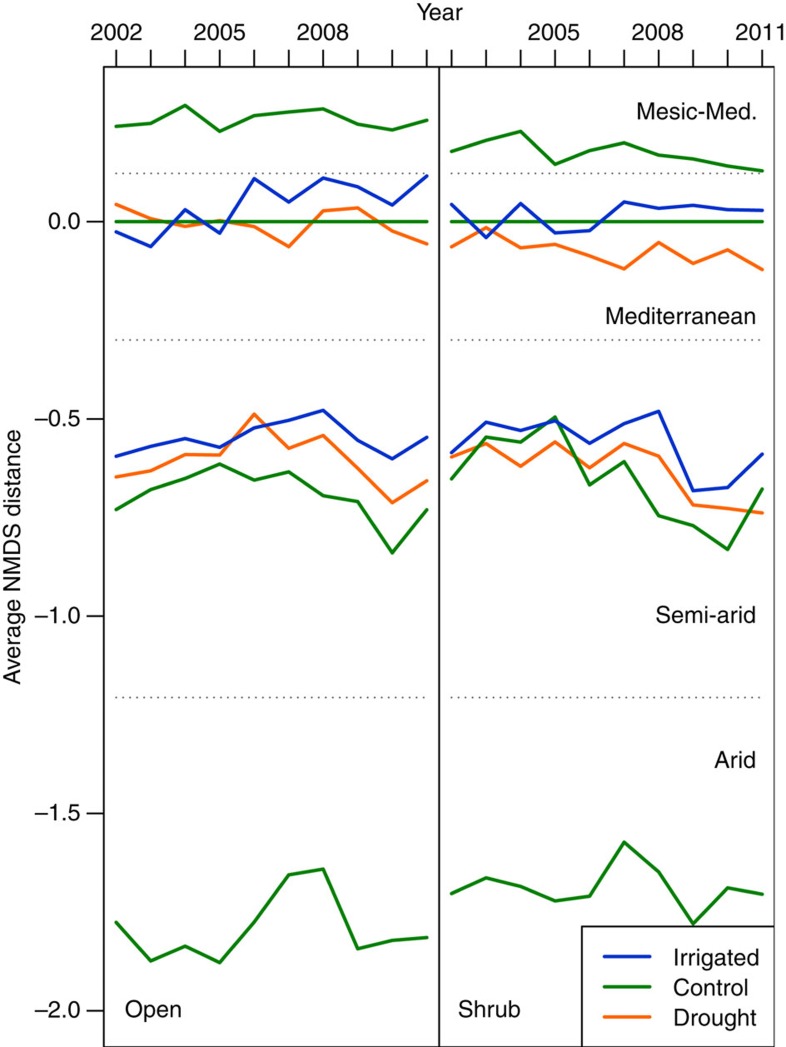
Summary of species compositional change given by non-metric multi-dimensional scaling analysis of a Jaccard dissimilarity matrix. The vertical axis corresponds to the climate gradient that runs from arid conditions in the South to mesic-Mediterranean conditions in the North. The proximity of lines along this axis corresponds to similarity in community composition. The first year is a baseline without manipulation where differences between treatments are due to natural spatial variation. The temporal trajectories of similarity in community composition are largely parallel for all treatments—differences among treatments did not change over time, confirming that there were no directional changes in community structure during 9 years of either artificial drought or irrigation. This was substantiated statistically using Redundancy analyses (RDA) on community structure: no interaction between the factors ‘year’ and ‘treatment’.

For visual illustration of the results we used a three-axis non-metric multi-dimensional scaling (NMDS) analysis performed on a ‘Jaccard’ dissimilarity matrix of total summed abundances within each treatment per site. For each year, sites per treatments were ranked, and Euclidean distances were calculated between NMDS values and averaged to display differences in values on one axis (Fig. 6). In addition, unconstrained principle component analyses plots for within SA and M patterns were done (Supplementary Fig. 2). Visual representation calculations were performed using R version 2.14.1 (‘vegan’ package^52^).

The univariate parameters biomass (log), density (log) and species richness were analysed by fitting Linear Mixed Models (LMM) for each site and microhabitat separately using the ‘LMM’ package within SAS software version 9.3. LMMs included the categorical fixed effects year and treatment, and the baseline values of 2001/2002 as a covariate. Plot intercepts were included as random error variables, to account for our replication over time. In addition, we identified some autocorrelation between our sample years and found that an autoregression (AR) function of lag 2 (years) generally provided the best fitting models, and so was included in all our LMMs. *Post hoc* contrasts between each treatment were assessed and corrected for multiple testing using Tukey–Kramer adjustments. Alternative models that included annual rainfall were tested (Supplementary Methods) but they had less explanatory power, though yielding similar results (Supplementary Tables 3 and 4).

Seasonal averages of soil moisture and temperature (Fig. 3) were assumed to be independent between years and were analysed with LMMs that included treatment, habitat and season year as fixed effects with sensor as random effect (model fitting and simplification protocol^53^). We performed model selection by AIC using both backward and forward stepwise search^54^. The full model included all main effects, two-way and three-way interactions. The results (Supplementary Table 1) present the most parsimonious model fitted with the restricted maximum likelihood method. The analyses were performed using R, version 2.15.0, and the nlme^55^ and MASS^54^ packages.

We also performed power analyses complemented with a survey of existing rainfall manipulation studies (Supplementary Methods). The analyses and the comparison with previous experiments indicated that ours is one of the most robust and comprehensive to date in terms of sampling effort (Supplementary Table 6), with a high probability of detecting a response to the manipulations.

## Author contributions

K.T., M.C.B. and J.M. contributed equally to this work. K.T., M.S., C.H. and J.K. conceived and designed the experiments. J.M., H.A.P. and I.K. performed the experiments: M.C.B., J.M., E.L.-T. and J.K. analysed the data. K.T., M.C.B. and J.M. wrote the paper, C.H., J.K., E.L. and M.S. provided editing.

## Additional information

**How to cite this article**: Tielbörger, K. *et al*. Middle-Eastern plant communities tolerate 9 years of drought in a multi-site climate manipulation experiment. *Nat. Commun.* 5:5102 doi: 10.1038/ncomms6102 (2014).

## Supplementary Material

Supplementary Information Supplementary Figures 1-2, Supplementary Tables 1-6, Supplementary Methods and Supplementary References

## Figures and Tables

**Table 1:** Statistical results of multivariate redundancy analyses (RDA) testing for treatment effects on species composition.

**RDA model**	**Semi-arid**		**Mediterranean**	
	***P-*****value**	**Trace (%)**	***P-*****value**	**Trace (%)**
*Open*				
Year	0.001	40.4	0.001	14.2
Year × treatment	0.13	1.6	0.45	1.5
				
*Shrub*				
Year	0.001	36.4	0.001	11.9
Year × treatment	0.32	1.6	0.081	1.9

Significant year × treatment interactions would indicate differences among treatments in their temporal dynamics, for example, increasing differences among treatments with time. The results indicate highly significant variations in community structure among years for both sites and microhabitats, but no effect of treatment on the temporal development. This is visualized in Fig. 6 (temporal trajectories) and in Supplementary Fig. 2 (corresponding principal component analysis for each site and microhabitat separately).
