# Supplementary material for: Middle-Eastern plant communities tolerate 9 years of drought in a multi-site climate manipulation experiment
Source: Nat Commun. 2014 Oct 6;5:5102. doi: 10.1038/ncomms6102 (PMC4205856; doi:10.1038/ncomms6102)
Supplement: Supplementary Information — Supplementary Figures 1-2, Supplementary Tables 1-6, Supplementary Methods and Supplementary References [file ncomms6102-s1.pdf]

a)

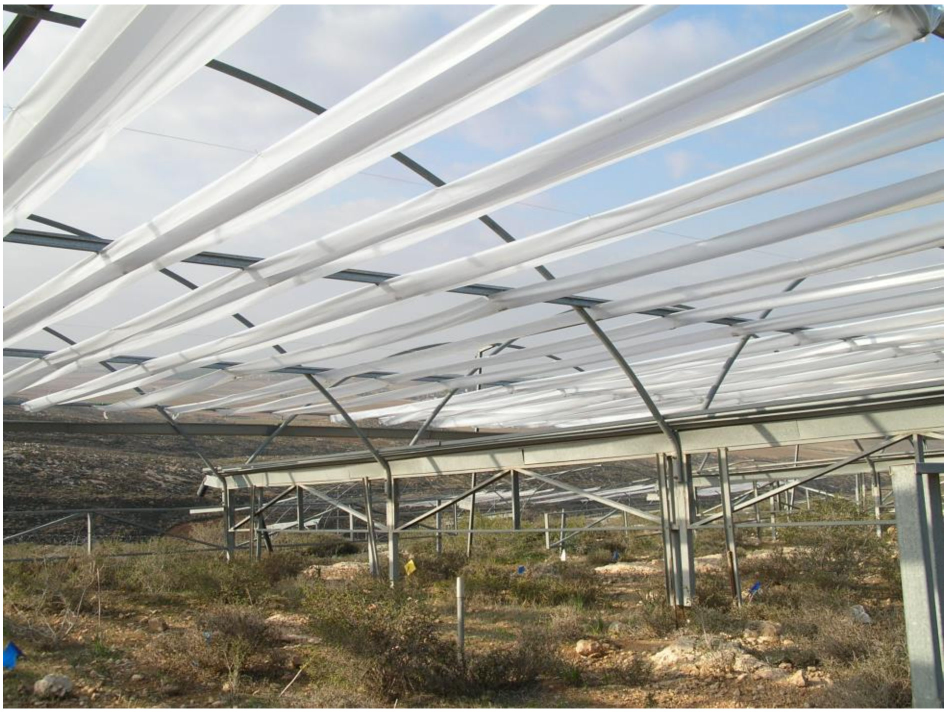

b)

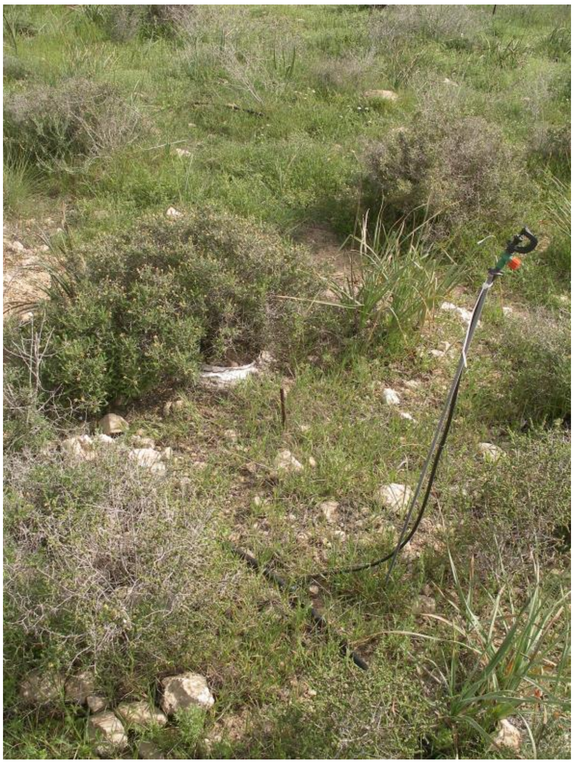

Supplementary Figure 1. Photographs of treatments. a) A view from below a rainout shelter in the semi-arid site with v-shaped plastic strips covering 30% of the area. b) A sprinkler in an irrigated plot in the same site at peak season.

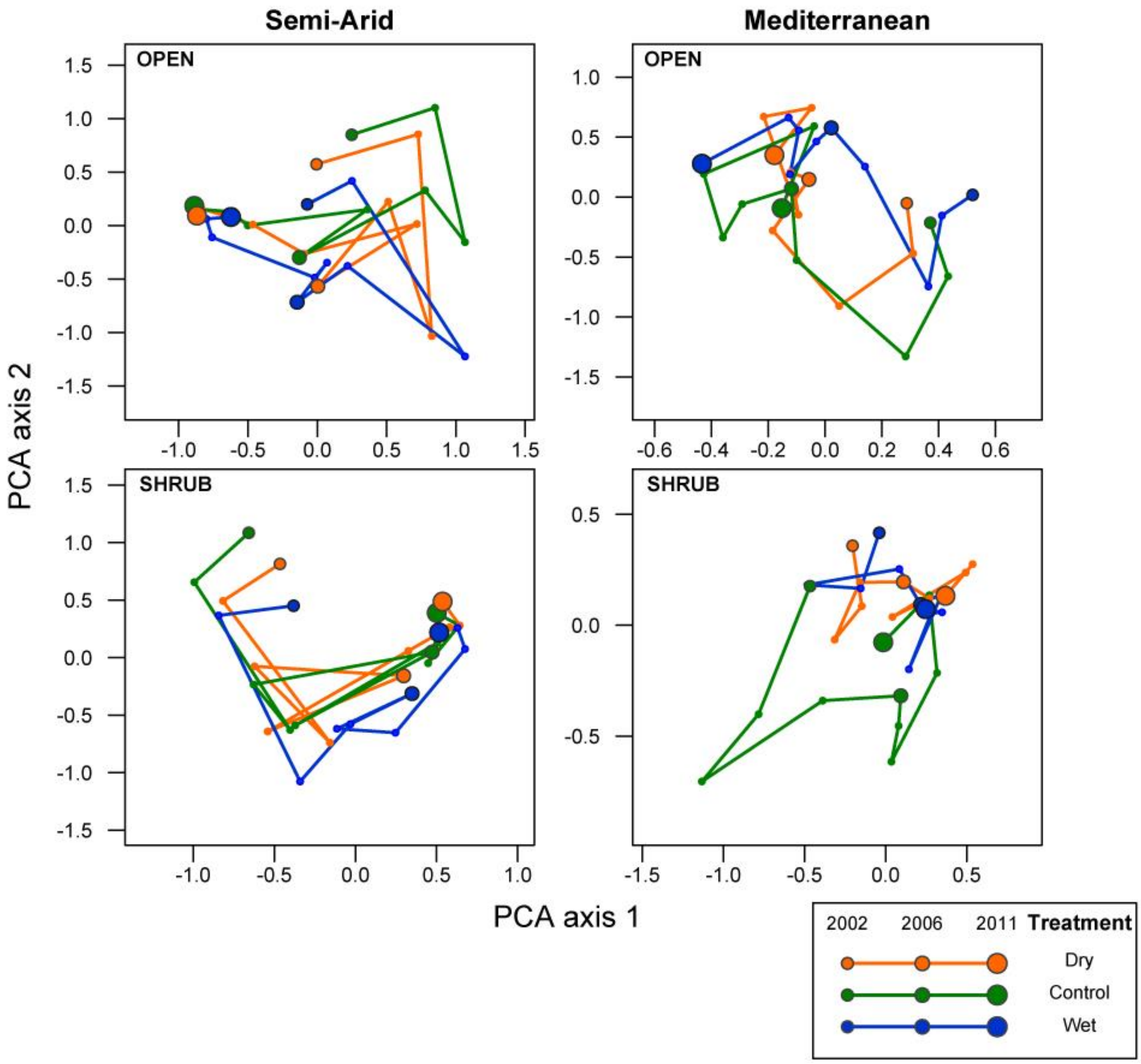

Supplementary Figure 2. Graphical representation (PCA) of the response of plant communities to nine years of rainfall manipulation. Data stem from two sites (semi-arid and Mediterranean) and two microhabitats (open areas between shrubs and under shrubs). The community trajectories in the two manipulated sites correspond to those of the NMDS in Fig. 6 in the main text. They show that trajectories of dry treatments (orange), wet treatments (blue) and controls (green) were largely parallel, i.e. the treatments did not affect the temporal development of plant communities (see Supplementary Table 2 for statistical results, namely nonsignificant treatment × year interactions from RDA analysis).

Supplementary Table 1. Summary of statistical results (F-values) for soil moisture (vol. %) and temperature (°C) measurements (Fig. 3). Linear mixed models for each parameter and site (Semi-Arid or Mediterranean) included treatment, (micro) habitat (under Shrubs *vs*. Open), Year and their interactions as fixed effects; sensor was included as a random effect. Unfortunately, theft, vandalism and temporary malfunctioning of the equipment led to occasional lack of data. Therefore, we analyzed the data for each site separately by averaging per day, month, and growing season. Only days with recordings for more than 21 hours, months with more than 15 days and years with four or more months of data were included in the analysis (94% of all months for temperature and 93% for soil moisture). The final data comprised of the years 2002-2004, and 2006-2009 for the SA site, and of the years 2003-2009 for the M site.

*p<0.05, **p<0.01, ***p<0.001; n.s. not significant.

		**Soil Moisture**				**Soil Temperature**			
**Fixed Effect**	**Df**	**Semi-arid**		**Mediterranean**		**Semi-arid**		**Mediterranean**	
Treatment	2	40.25	***	8.41	*	15.27	***	30.28	***
Habitat	1	7.35	**	2.57	n.s.	0.62	n.s.	0.42	n.s.
Year	6	80.69	***	59.46	***	498.14	***	410.25	***
Treat * Habitat	2	0.45	n.s.	0.62	n.s.	13.51	**	2.30	n.s.
Treat * Year	12	74.90	***	18.28	n.s.	89.48	***	48.80	***
Habitat * Year	6	4.88	n.s.	12.78	*	15.61	*	65.74	***
Treat * Habitat * Year	12	17.38	n.s.	16.02	n.s.	116.05	***	71.54	***

Supplementary Table 2. Statistical table of results for univariate parameters biomass, density and species richness (Fig. 5). Linear Mixed Models for each parameter, site (Semi-Arid or Mediterranean) and micro-habitat (Open or under Shrubs), with categorical fixed factors for Year, Treatment and their interaction, plus Baseline (2001/02) data used as a covariate. Treatment effects indicate overall differences between wet, dry and control manipulation treatments, with significant results shown by asterisks (*p<0.05; ** p<0.01; *** p<0.001), and significant Tukey-Kramer adjusted contrasts for post-hoc pair-wise analysis between individual treatments (W = wet; D = dry; C = control). Note that no pairwise contrasts among treatments are significantly different after a Benjamini & Hochberg[1] correction for false discovery rate.

		**Semi-Arid**				**Mediterranean**			
	Type III Effects	DF	F-Value	contrast		DF	F-Value	contrast	
	**Biomass**								
Open	Baseline(2002)	1,11	14.39	**		1,11	19.78	**	
	Year	7,84	17.37	***		7,84	9.01	***	
	**Treatment**	2,11	3.02	ns		2,11	2.00	**ns**	
	Year*Treatment	14,84	1.30	ns		14,84	3.12	**	
Shrub	Baseline(2002)	1,11	1.03	ns		1,11	5.88	*	
	Year	7,84	18.28	***		7,84	5.78	***	
	**Treatment**	**2,11**	**4.85**	*	**W>C**	2,11	1.11	ns	
	Year*Treatment	14,84	1.30	ns		14,84	1.71	ns	
	**Density**								
Open	Baseline(2002)	1,11	6.82	*		1,11	4.47	*	
	Year	8,96	60.22	***		8,96	44.11	***	
	**Treatment**	2,11	2.44	ns		**2,11**	**4.48**	*	**C>D**
	Year*Treatment	16,96	4.07	***		16,96	2.18	*	
Shrub	Baseline(2002)	1,11	0.37	ns		1,11	3.09	ns	
	Year	8,96	59.69	***		8,96	24.54	***	
	**Treatment**	2,11	0.70	ns		2,11	0.37	ns	
	Year*Treatment	16,96	3.11	**		16,96	2.73	**	
**Species Richness**									
Open	Baseline(2002)	1,11	0.00	ns		1,11	16.11	**	
	Year	8,96	48.79	***		8,96	35.41	***	
	**Treatment**	**2,11**	**5.29**	*	**D>C**	2,11	1.89	ns	
	Year*Treatment	16,96	3.00	***		16,96	1.95	*	
Shrub	Baseline(2002)	1,11	0.26	ns		1,11	26.56	***	
	Year	8,96	79.26	***		8,96	20.48	***	
	**Treatment**	2,11	0.29	ns		2,11	0.83	ns	
	Year*Treatment	16,96	3.95	***		16,96	1.26	ns	

Supplementary Table 3. Additional statistical analyses with year included as a continuous variable (a "treatment x year" interaction would show treatment effects) and rainfall at T0, T-1 and T-2 as predictor variables (see Supplementary Methods 1.5 below for explanation). Although not directly comparable, this analysis has less explanatory power than the main analysis (Supplementary Table 2 and main text), and the main message of 'negligible treatment response' stays the same. F-values are shown for the "Treatment x Year" interaction. Significant P-values are shown using asterisks: *<0.05 **<0.01. In addition, directions of significant contrasts are shown highlighting which treatments may differ: D=droughted; C=Control; W=Irrigated. Results highlighted in red are statistically significant results that are opposite to the predicted direction. Using the Benjamini & Hochberg correction[1] for multiple testing (16 tests), only SA open density W>C remains significant.

Dependent variable	*Treatment X year*	Semi-Arid			Mediterranean		
		F-value	contrasts	AIC	F-value		AIC
Density	Open	7.295	** W,D>C	225.28	2.930		134.82
	Shrub	0.749		289.37	1.300		162.15
Sp. Richness	Open	2.942		643.40	0.545		647.14
	Shrub	0.361		690.41	0.382		596.51
Biomass	Open	2.939		227.80	0.823		169.33
	Shrub	5.987	* W>C	266.20	1.610		242.17

Supplementary Table 4. Additional statistical analyses including rainfall at T0, T-1 and T-2 as predictor variables (see Supplementary Methods 1.5 below for explanation). In this analysis, we excluded year as an explanatory variable and only used annual precipitation. The analysis has less explanatory power than the main analysis (Supplementary Table S2 and main text) and the main message of 'negligible treatment response' stays the same. F-values are shown for "Treatment" effects. Significant P-values are shown using asterisks: *<0.05. In addition, directions of significant contrasts are shown highlighting which treatments may differ: D=droughted; C=Control; W=Irrigated. Results highlighted in red are statistically significant results that are opposite to the predicted direction. No results are significant after a Benjamini & Hochberg[1] multiple testing correction, based on 16 hypothesis tests.

Dependent variable	*Treat-ment*	Semi-Arid			Mediterranean		
		F-value	contrasts	AIC	F-value		AIC
Density	Open	1.362		224.87	3.645		116.27
	Shrub	1.139		266.41	0.628		138.10
Sp. richness	Open	7.080	** C<D;W	598.83	1.745		566.29
	Shrub	0.544		628.20	1.126		523.79
Biomass	Open	3.941	* W>C	213.51	0.654		168.55
	Shrub	6.581	* W>C	249.99	0.601		225.98

Supplementary Table 5. List of common annual plant species along the climate gradient as observed in ten study years in permanent quadrats. The table illustrates the extraordinarily high species richness of the systems: 123 annual species were sufficiently frequent in the quadrats to be included in the analyses, but more than 600 species were found in the research sites. The overlapping species pool is illustrated by arranging the species according to their occurrence from South to North. The shared species pool is a prerequisite for our approach of testing BEM predictions because we do not assume migration as in BEM (dispersal of our component species is negligible[2]) but more realistic *in situ* changes of the plant communities in response to the treatments. Relatives and progenitors of globally important cereals and legumes are in bold. All grasses are very common or even dominant (*Avena* and *Hordeum*) in the studied communities. Nomenclature follows Feinbrun-Dothan[3].

**Species**	**Arid**	**Semi-Arid**	**Mediterranean**	**Mesic-Med.**
Diplotaxis harra	X			
Gymnarrhena micrantha	X			
Lappula spinocarpos	X			
Plantago ovata	X			
Reboudia pinnata	X			
Schismus arabicus	X			
Adonis dentata	X	X		
Astragalus tribuloides	X	X		
Carrichtera annua	X	X		
Filago desertorum	X	X		
Herniaria hirsuta	X	X		
Plantago coronopus	X	X		
Reichardia tingitana	X	X		
Picris damascena	X		X	
Helianthemum salicifolium	X	X	X	
Stipa capensis	X	X	X	
Erodium laciniatum	X		X	X
Euphorbia chamaepeplus	X	X	X	X
Hippocrepis unisiliquosa	X	X	X	X
Urospermum picroides	X	X	X	X
Aegilops kotschyi		X		
Cichorium pumilum		X		
Crithopsis delileana		X		
Onobrychis christa-galli		X		
Silene apetala		X		
Trigonella stellata		X		
Trisetaria macrochaeta		X		
Ainsworthia trachycarpa		X	X	
Calendula arvensis		X	X	
Callipeltis cucullaria		X	X	

Chaetosciadium trichospermum	X	X	
Clypeola jonthlaspi	X	X	
Daucus subsessilis	X	X	
Diplotaxis viminea	X	X	
Erodium ciconium	X	X	
Galium murale	X	X	
Helianthemum ledifolium	X	X	
Minuartia picta	X	X	
Onobrychis caput-galli	X	X	
Onobrychis squarrosa	X	X	
Ononis ornithopodioides	X	X	
Ononis sicula	X	X	
Pimpinella cretica	X	X	
Pterocephalus brevis	X	X	
Sedum pallidum	X	X	
Silene colorata	X	X	
Silene nocturna	X	X	
Silene oxyodonta	X	X	
Aegilops peregrina	X	X	X
Alyssum strigosum	X	X	X
Anagallis arvensis	X	X	X
Atractylis cancellata	X	X	X
Avena barbata	X	X	X
Avena sterilis	X	X	X
Biscutella didyma	X	X	X
Brachypodium distachyon	X	X	X
Bromus fasciculatus	X	X	X
Campanula hierosolymitana	X	X	X
Catananche lutea	X	X	X
Catapodium rigidum	X	X	X
Convolvulus siculus	X	X	X
Coronilla scorpioides	X	X	X
Crepis sancta	X	X	X
Erodium gruinum	X	X	X
Erodium malacoides	X	X	X
Filago contracta	X	X	X
Filago palaestina	X	X	X
Filago pyramidata	X	X	X
Galium cassium	X	X	X
Galium judaicum	X	X	X
Geropogon hybridus	X	X	X
Hedypnois rhagadioloides	X	X	X
Hordeum spontaneum	X	X	X
Hymenocarpos circinnatus	X	X	X
Lagoecia cuminoides	X	X	X
Linum pubescens	X	X	X
Linum strictum	X	X	X

Lolium rigidum	X	X	X
Lotus peregrinus	X	X	X
Ononis reclinata	X	X	X
Parapholis incurva	X	X	X
Picris galilaea	X	X	X
Plantago afra	X	X	X
Plantago cretica	X	X	X
Psilurus incurvus	X	X	X
Pterocephalus plumosus	X	X	X
Rhagadiolus stellatus	X	X	X
Rostraria cristata	X	X	X
Scabiosa palaestina	X	X	X
Scorpiurus muricatus	X	X	X
Senecio vernalis	X	X	X
Theligonum cynocrambe	X	X	X
Thlaspi perfoliatum	X	X	X
Torilis leptophylla	X	X	X
Torilis tenella	X	X	X
Trifolium campestre	X	X	X
Trifolium clusii/bullatum	X	X	X
Trifolium purpureum	X	X	X
Trifolium scabrum	X	X	X
Trigonella monspeliaca	X	X	X
Valantia hispida	X	X	X
Valerianella vesicaria	X	X	X
Velezia rigida	X	X	X
Ziziphora capitata	X	X	X
Alopecurus utriculatus		X	
Cicer pinnatifidum		X	
Euphorbia oxyodonta		X	
Geranium rotundifolium		X	
Isatis lusitanica		X	
Mercurialis annua		X	
Ononis pubescens		X	
Silene palaestina		X	
Stachys neurocalycina		X	
Trifolium dasyurum		X	
Briza maxima		X	X
Convolvulus pentapetaloides		X	X
Crupina crupinastrum		X	X
Linum corymbulosum		X	X
Linum nodiflorum		X	X
Sherardia arvensis		X	X
Trifolium pilulare		X	X
Trifolium resupinatum		X	X
Trifolium stellatum		X	X

Supplementary Table 6. Sampling effort survey of previous rainfall manipulation experiments. Our study was compared with 12 important and large climate change experiments (16 publications) worldwide regarding duration and sampling effort. The focus is on experiments reporting community parameters similar to those in our study. Publications sharing the same number report from the same experiment. Note the highly varying intensity of applied treatments and differing ecosystems and growth forms. We highlight two measures, 'total replication' and 'total sampling effort' for assessing sampling effort and thus potential power of experimental designs but other measures can be easily obtained from the table. 'Total sampling effort' accounts also for the number of subsamples that researchers deemed most adequate for their study system. Note that the table is no exhaustive literature survey, and whilst we did our best to extract the correct sampling scheme from the method descriptions, there may be cases of misinterpretation.

			**Replication**						**Treatments**	**Response variables**				**dominant life form**	**Comments**
	**Study [Ref. No.]**	**location**	**manipu-lated years**	**replicates per treatment[1]**	**sampling units per replicate**	**area per sampling unit**	**total replica-tion[2]**	**total sampling effort[3]**		**biomass**	**richness/ diversity[4]**	**density**	**community composition[5]**		
**Rainfall manipulation experiments**															
	this study	(arid) semi-arid & Med. Israel	9	5 x 2 sites + 2 control sites	5 x 2 micro-habitats	0.04m²	**45 (x 2 sites + 2 control sites)**	**450 (x 2 sites + 2 control sites)**	3 (+30%, -30% rain; control)	no drought response, 1 response out of 16 to irrigation	no overall response, marginal increase under drought in 1 of 16 cases	no overall response, marginal decrease under drought in 1 of 16 cases	no response	native annuals	ten years = nine manipulated generations, high species richness, pre-treatment data available
1a	Zavaleta et al. 2004 [4]	Med. California, USA	3	8 - 64, dep. on treatment	1	0.78m²	**24 - 172**	**24 - 192**	8 (+50% rain; +1°C; +300ppm CO_2; +N; combinations)		marginal effect of 3 treatments (<1species change)			exotic annual grasses and forbs, native perennials	results reported only for last year; moderate richness of 10spp/0.78m²
1b	Shaw et al. 2002 [5]	Med. California, USA	3	6 - 48, dep. on treatment	1	0.014 m²	**18 - 144**	**18 - 144**	8 (+50% rain; +1°C; +300ppm CO_2; +N; combinations)	+ CO_2 reduced biomass but only with other factors				exotic annual grasses and forbs, native perennials	method interpretation amended using Dukes et al. 2005 (5); (focus on CO_2)
1c	Dukes et al. 2005 [6]	Med. California, USA	5	8 - 64, dep. on treatment	1	0.014 m²	**40 - 320**	**40 - 320**	8 (+50% rain; +1°C; +300ppm CO_2; +N; combinations)	only +N treatments had consistent (positive) effect				exotic annual grasses and forbs, native perennials	Intense +N treatment: 14x of natural deposition; biomass incl. below ground

2	Suttle et al. 2007 [7]	Med. California, USA	5	6	2 (richness) 6 (biomass)	0.25m^2 (richness) 0.09m^2 (biomass)	**30**	**60 / 180**	3 (increase of Winter or Spring rain, control)	strong increase by Spring irrigation only	strong decrease by Spring irrigation only			annual grasses and forbs	treatments: +20% of annual mean added in 3 months (Winter or Spring); no pre-treatment data
3	Harpole et al. 2007 [8]	semi-arid California, USA	1	5	1	0.113m^2	**5**	**5**	4 (+30% rain; +10gN/m^2; +rain & N; control)	increased by combined +N & + rain treatment	decreased by +N and +N & +rain treatment			exotic annual grasses and forbs	highly pulsed treatment: +30% annual rain added during l2 months; +10gN/a
4	Miranda et al. 2011 [9]	semi-arid Spain	5	4	1	0.035m^2	**20**	**20**	4 (ambient or -30% annual rain, with/no changed distribution)	decrease in 1 out of 4 treatments in last year				not reported	
5	Miranda et al. 2009 [10]	semi-arid Spain	1	15 x 3 sites	1	0.08 m^2	**45**	**45**	9 (3x3 combination of ambient, -25%, -50% rainfall; weekly, biweekly, or monthly shots)	reduced by only 1 treatment (-50%drought), no effect of frequency	reduced by only 1 treatment (-50%drought), no effect of frequency	reduced by only 1 treatment (-50% rain), no effect of frequency		annuals	3 locations with 1 full-cover rainout shelter; single treatments applied manually
6a	Prieto et al. 2009 [11]	Med. Spain	7	3	5	61 point needles	**21**	**105**	3 (drought, +0.7°C warming, control)		decreased by drought, no effect of warming		increasing dissimilarity of drought *vs.* control; no warming effect	shrubs	drought treatment extended summer drought by 2-3 months, study during post-fire succession
6b	Lloret et al. 2009 [12]	Med. Spain	7	3	5	61 point needles	**21**	**105**	3 (drought, +0.7°C warming, control)				increasing dissimilarity of drought *vs.* control; no warming effect	shrubs	drought treatment extended summer drought by 2-3 months, study during post-fire succession
7	Peñuelas et al. 2007 [13]	six countries from North-South Europe	1-7 (site-specific)	3 x 6 sites	1 - 5 (site-specific)	point needles, site-specific	**72 richness; 75 biomass**	**243 richness; 261 biomass**	3 (drought, +0.7°C warming, control)	Marginally sign. responses to drought and warming in only 1 site	decreased by drought in only 2 of 6 sites; no effect of warming			shrubs	sampling effort varied among sites and variables, extracted from figures, partial pre-treatment data
8	Evans et al. 2011 [14]	semi-arid Colorado, USA	11	2	5	0.25m^2	**22**	**110**	3 (-50%, -75% of growth season precipitation, control)	-75% treatm. lower than control in later years, marginal effects of -50%	Only -75% treatm. response (lower than control in 3 years)		ruderals had larger response	perennial grasses, shrubs, with ruderals	Sign. treatment effects only after 4 years or later, % cover reported as proxy for biomass, no pre-treatment data
9a	Grime et al. 2000 [15]	temperate UK	5	5 x 2 sites	1	0.25m^2 (biomass), 25 point needles (comm. compos.)	**50**	**50**	6 (drought, +20% irrigation, +3°C winter warming, and combinations, control)	marginal effects in unproductive site, strong effects in productive site			marginal effects in unproductive site, strong effects in productive site	perennial graminoids and forbs	methods taken from Grime et al. 2008 (15); drought treatment blocked rainfall completely in main growth season

9b	Grime et al. 2008 [16]	temperate UK	13	5	1 (biomass, commun. composit.); 4 (richness)	$0.25m^2$ (biomass), $0.01m^2$ (richness), 25 point needles (communnity structure)	**65**	**65 / 260**	6 (drought, +20% irrigation, +3°C winter warming, and combinations, control)	reduced by only 1 treatment (drought)	minor reduction by drought treatment		marginal effects of drought treatments	perennial graminoids and forbs	overall little treatment effects; drought treatment blocked rainfall completely in main growth season July-August
10	Báez et al. 2013 [17]	semi-arid New Mexico, USA	4 and 5	3 x 3 sites	3 transects with 10 units each	$1m^2$	**12 and 15**	**360 and 450**	3 (+42%, -50% rain; control)		no consistent effects	cover: species- and site-specific, dominant grass affected in 2 sites	significant effect of drought and irrigation on subdominant community	shrubs, perennial grasses or both	p was set at 10%, no pre-treatment data
11	Collins et al. 2012 [18]	temperate Kansas, USA	19	1 x 2 habitat types	10 sampling units within one transect	$10\ m^2$	**19**	**190**	2 Control; + highly variable amount of water added (6%-56%)		no effect	cover increased with irrigation in one habitat		C4-grasses with some C3 forbs	no pre-treatment data, pre-treatment burning regime different
12	Yang et al. 2011 [19]	Temperate steppe, Inner Mongolia, China	6	4	1	$1m^2$	**24**	**24**	4 (+30% rain; +10gN/m²; +rain & N; control)		unclear: significant treatment effect but no interaction with year			perennial grasses and forbs	Highly pulsed treatments: +30% annual rain added during l2 months of growth season; +10gN/a applied in one pulse. No pre-treatment data

[1] True statistical replicates per treatment.
[2] Number of replicates multiplied with manipulated years.
[3] Number of sampling units observed per treatment during the entire study period (replicates x manipulated years x # of subsamples). This number represents the total sampling effort.
[4] Includes reports on species number (richness) or diversity indices (e.g. Shannon, Simpson).
[5] Only composition analyses at multi-species level were considered (multivariate analyses), not reports on functional types.

1. Supplementary Methods

1.1 Detailed site description

Our experiments were conducted at four research sites along the steep climatic gradient that runs from the Negev desert to the upper Galilee in Israel (Fig. 1; Fig. 2). The study region is characterized by particularly high plant species richness, most of which is accounted for by annual plants.

The Arid site (A), located 3 km north of Sde Boqer in the central Negev desert (N 30°52' E 34°46'; 470m a.s.l.), is the southernmost station of this gradient[20]. This site receives 90mm mean annual precipitation, with high inter-annual variation (CV = 51%), and lies on desert lithosol. The area is characterized by open vegetation, with scattered small shrubs (*Artemisia sieberi* and *Zygophyllum dumosum*) that cover less than 5% of the area and are often dead. Annual plant cover is scarce (0-15%) and varies strongly with annual rainfall. In this site, annuals tend to grow in higher densities and attain larger biomass under shrub canopies and in small depressions and wadis. Annuals account for more than 95% of the species and up to 100% of the annual primary production (ANPP[21,22]), with a higher percentage in drier areas and areas with larger grazing pressure.

The semi-arid site is located approximately 20km north of Beer Sheva near Lahav (N 31°23' E 34°54', 590m a.s.l.). It has an average annual rainfall of approximately 300mm with a CV for inter-annual variation of 37% and the soil is a loess lithosol. The vegetation is dominated by annuals which cover, depending on rainfall, between 10% and 25% of the area. Shrubs - mostly the dwarf shrub *Sarcopoterium spinosum*- are common and cover approx. 25%. The spatial pattern of annuals varies among years, and they tend to be less abundant under shrubs in dry years but exhibit no difference between microhabitats in wet years. Annuals include approx. 90% of all species in that region and herbaceous plants, most of which are annuals, account for 80-90% of the annual primary production.

The Mediterranean site near Matta is situated in the Judean mountains approximately 15 km southwest of Jerusalem (N 31°42' E 35°3'; 620m a.s.l.). It receives an average of 540mm annual precipitation (CV=30%) and lies on Terra Rossa

soils. The vegetation consists of dwarf shrubs (dominated by *Sarcopoterium spinosum*) that cover approx. 40% of the area and annuals growing mostly in open areas and partly beneath shrubs. Annuals cover up to 90% of the open areas and include approx. 80% of all species. Herbaceous plants (mostly annuals) account for 50% (ungrazed areas) to 90% (normally grazed to overgrazed areas) of the ANPP.

The Mesic-Mediterranean site, located approximately 10 km East of Nahariyya near Ein Ya'akov in the northern Galilee (N 33°0' E 35°14'; 500m a.s.l.) is the northernmost site of the gradient. With a mean annual precipitation of 780mm and the smallest inter-annual variation (CV = 22%), this site stands over montmorillonitic Terra Rossa soils. The vegetation is best described as a closed shrubland (Mediterranean maquis/garrigue) dominated by the shrubs *Sarcopoterium spinosum* accompanied by *Calycotome villosa* and *Cistus* spp., and scattered small trees species such as *Quercus calliprinos* Webb that together cover up to 70% of the area. Annuals cover most of the remaining open area (total cover 90-100%) and comprise approx. 80% of all species. Between 50% (ungrazed areas) and 90% (grazed areas) of the ANPP can be accounted for by herbaceous plants, though shrubs and small trees may attain a large fraction of the standing biomass.

1.2 Detailed description of rationale and procedure of climate manipulations

The intensity and direction of our treatments was inspired by regional climate scenarios that were developed through statistical and dynamic downscaling of global models[23,24]. Downscaling is particularly important for the study region because rainfall varies considerably across very small distances (Fig. 1a, Fig. 2). At the onset of the study, we depended on low-resolution global circulation models (GCMs[25]). Though suggesting a more likely decrease in mean annual precipitation, they had a high uncertainty and ranged from a 30% decrease to a 30% increase. To cover this range of possibilities, we applied both drought and irrigation. The most recent ensembles of downscaled climate scenarios, which were produced alongside our study, predict an increasing aridification throughout most of the study region, including a decrease of the annual precipitation with regional variation and an increase in temperatures[23,24]. The average predicted decrease in rainfall is roughly 20%, with large geographic variation (approx. 10-30%). Therefore, the drought treatment is the most relevant for

predicting climate change response in our study systems. Since temperatures were also increased by that treatment (Fig. 3, Supplementary Table 1) it mimicked the predicted change even more realistically, making our findings particularly robust.

Details about the rationale of the drought treatment can be found in the methods section of the main text. The method of using permanent strips (Supplementary Fig. 1) instead of a closed roof to exclude rainfall was chosen mainly because it has been shown that the strips do not produce unwanted side effects, such as shading, reduction of temperature or reduction of wind speed, all of which would counteract the intended treatment effect[26]. In order to confirm this assumption also in our study, we measured soil moisture and temperatures as described in the methods in the main text. The findings from our on-site environmental recordings (Fig. 3, Supplementary Table 1), confirm the effectiveness of both the drought as well as the irrigation treatment, i.e. they had the desired effect on water availability and temperature.

The general rationale of the irrigation treatment (Supplementary Fig. 1) was similar to that of most previous irrigation manipulation experiments[e.g. 17] in that irrigation was increased based on the long-term average to make sure that irrigation would lead to a detectable increase in water availability compared to the long-term average, irrespective of precipitation variation among years. The solution was to supplement an amount which is consistent with the 'positive' scenarios suggested by our climatologist colleagues, and within the range of low resolution GCMs at the onset of the study; i.e. we added 30% of the long-term average rainfall to the irrigated plots. This resulted in an additional 164mm rainfall in the Mediterranean and 90mm in the semi-arid site. We carefully checked the rainfall distribution at the sites in the past years to identify a protocol that fulfilled the following criteria: 1) irrigation should be spread across the entire season as to be largely parallel to the drought treatment; 2) irrigation should take place immediately after significant rain events and under cloudy sky so not to alter rain frequency and distribution and to minimize evaporation loss; 3) the irrigation should not extend the growing and monitoring season, i.e. the last irrigation should be applied no later than mid-April. To meet these criteria, we supplemented 10mm of rain after each major rainstorm that exceeded 5mm, with the

final supplementary irrigation occurring usually in mid-March, while in two seasons (2007/08 and 2008/09) irrigation ended mid-April.

Generally, in most years the irrigation was similar to the initially proposed +/- 30% scenarios. Namely, the irrigation amounted to the following increase relative to ambient annual rainfall in each year (chronologically from 2002/03 to 2010/11): 121%, 136%, 129%, 132%, 127%, 139%, 143%, 132%, and 147% respectively, for the Mediterranean, and 126%, 138%, 124%, 146%, 134%, 149%, 168%, 137%, 162%, respectively, for the semi-arid site. Interestingly, years with larger deviations from the +30% scenario did not show larger effects of irrigation in the response variables (and *vice-versa*), indicating that the irrigation per se was more important than the exact proportional increase.

1.3 Calibrating biomass measurements

The calibrating destructive measures for above-ground biomass were taken from open patches by sampling biomass of herbaceous vegetation in randomly placed 20cm × 20cm quadrats at peak season (end March to mid-April). A total of 50 samples per treatment (10 per plot) were collected each year. Plants were cut just above soil surface, bagged separately for each quadrat, oven-dried at 70°C in a forced air oven for 48hs and weighed. The data correlated significantly with the undestructive measures used in the main analyses (see main text), and additional analyses on the data from the harvest indicated similar resistance to the climate manipulations (Kigel et al. unpublished data).

1.4 Assessing experimental power

We acknowledge that larger sampling effort is always desirable in any experiment, particularly in ecology. However, determining the strength of our experiment both in terms of statistical power and contrasting to other experiments of similar type can maybe go some way to justifying our main suggestion that highly variable, water limited systems are more resistant to climate change than expected.

Supplementary Table 6 presents a qualitative comparison between the experimental design of our study and the strongest relevant climate change

experiments we are aware of, namely rainfall manipulation experiments which measured plant community responses. None of the chosen parameters are ideal for evaluating power, but in combination a relatively consistent picture emerges. The comparison (Supplementary Table 6) shows that our experiment compares with the most comprehensive studies of its kind reported to date in terms of sampling effort: number of sites; number of years; number of samples; and number of species, and it encompasses the largest number of plant generations ever observed. Three experiments report more years of climate change manipulation than our study, and these were conducted at single sites and with long-lived species, or they exhibited lower sampling effort. The power of our experiment is further amplified by extraordinarily high species richness (absolute and relative), high plant density and short plant lifespan of the focal plants, i.e. even minor effects (e.g. on few species) should have amplified during nine years of study via a positive feedback between fecundity and species density. The fact that other studies yielded significant community responses with lower sampling effort (Supplementary Table 6) further corroborates our claim of high resistance of our study systems.

We also ran simulated post-hoc power analyses using the variances contained within the data collected (analyses performed in R-version 2.14.1). Simulations were run for all 12 univariate analyses performed for each response variable (density, species richness, biomass) in each site (Semi-Arid, Mediterranean) and microhabitat (open, sub-shrub). Means and standard deviations were calculated across the 5 plots within each treatment in each year. A mean of the 3 treatments was then taken. 5 random values were then drawn from a normal distribution using these values (simulated control treatment), and a further 5 values were drawn with proportionally reduced mean values (simulated dry treatment). 2002 values were drawn from normal distributions with the same means. A stochastic algorithm was used to alter the ranks of plots drawn for each year to create the 'Random effect' variance (and fitted to the outputs from our analysis). For each realisation the same mixed model analysis as performed in the article was applied (see section 'Data Analyses section': Fixed effects: Data = 2002 + Year + Treatment + Year x Treatment, Random effects: Plot intercepts). 10,000 realisations were run for each proportional effect size, which

ranged from 0.5% to 60% decrease in plant responses. 80% power values were then extrapolated for each response variable and dataset, i.e. the proportional change between two treatments that would lead to 80% of samples drawn from these variances being significant at the alpha value of 0.05.

The results from the power analysis reflect the variance structure for each response variable. In general, variance is lowest for species richness, then density and then biomass, hence our results show that significant effects are likely to be detectable for proportional treatment effect sizes ranging between: for species richness (±18% to 26%), density (±27% to 39%), and biomass (±38% to 51%). These values fit fairly well against our actual mixed model results, whereby our few significant effects showed changes greater than these limits (e.g. density in M open was 46% lower in dry than control; Fig. 5, Supplementary Table 2), and most non-significant results showed less change than 'needed'.

It should be noted though that post-hoc power analyses are not well advocated[27,28]. Part of the problem is determining thresholds for effect sizes of what we could reasonably expect to see. However, by comparing these proposed effect sizes with the observed response variables both across sites (Fig. 4) and among years at a site (Fig. 5), suggests that our experimental design was probably strong enough to find appropriate responses if they existed. Although rainfall difference is bigger between sites than for our applied manipulations, the proportional change in response variables are also much larger (Fig. 4). Among year within site differences also suggest that at least phenotypic, if not selection, responses should be observable over many years (e.g. SA average fold differences between driest and wettest years in open plots: richness = 2.1, density 11.6, biomass 4.3). We therefore deduce that other effects, possibly specific plant adaptations to variability, are likely to be slowing down any community responses to the climate manipulations.

1.5 Selection of model for testing treatment effects

Our core prediction was that communities will change their structure due to climate change, and we have included the factor 'time' as years in the models. However, one may argue that precipitation in the study years should have been included in the

models. For example, there is a large amount of environmental variation between years which could have been caused by precipitation differences. We felt it best to capture this by including “year” as a categorical variable (Supplementary Table 2) – thereby not making any assumptions on precisely which (combination) of several possible environmental variables were causing the between-year variations in community parameters.

Including precipitation in the statistical models did not change our finding of ‘no response to the treatments’ – despite containing far fewer parameters, models that included precipitation even had a larger AIC than those described purely by categorical years (Supplementary Table 3 and 4).

Nevertheless, we had thought of including rainfall within the statistical model. It is not possible to include rainfall when year is a categorical variable, but we did try it with year as a continuous variable (focusing on a “treatment x year” interaction to show us any manipulation effects, Supplementary Table 3). Analogous to the Auto Regressive (lag 2) function used in the main analysis, we found a model which included rainfall in year T (current growing season) T-1 (year previous to growing season) and T-2 (2 years prior to growing season) to have the best fit. We have also tested our response parameters by removing “year” completely from the model, and including rainfall at T, T-1 and T-2 instead (Supplementary Table 3). Supplementary Tables 3 and 4 show that in general we find that while there are some subtle differences in details, the overall message is the same irrespective of the analyses, i.e. very few significant changes in the community due to the experimental treatments. More specifically, when rainfall was included in a statistical model as a predictor, treatment responses often explained some part of the remaining variance – as shown by the generally higher F-values (Supplementary Tables 3 and 4 compared to 2), but still only showed three significant responses, and never any difference between wet and dry.

Therefore, to keep the manuscript concise, we base our conclusions on the original and most powerful methods presented in the main text and in Supplementary Table 2.

Supplementary References

1. Benjamini, Y. & Hochberg, Y. Controlling the false discovery rate: a practical and powerful approach to multiple testing. *J. Royal Stat. Soc*. B, **57**, 289-300 (1995).
2. Siewert, W. & Tielbörger, K. The dispersal dormancy trade-off in annual plants: putting model predictions to the test. *Am. Nat.* **176**, 590-600 (2010).
3. Feinbrun-Dothan, N., Danin, A. *Analytical Flora of Eretz Israel* (Cana Publishing House, Jerusalem, 1991).
4. Zavaleta, E. S. *et al.* Additive effects of simulated climate changes, elevated CO_2, and nitrogen deposition on grassland diversity. *Proc. Natl. Acad. Sci. USA* **100,** 7650-7654 (2003).
5. Shaw, M. R., *et al.* Grassland responses to Global Environmental Changes suppressed by Elevated CO_2. *Science* **298**, 1987-1990 (2002).
6. Dukes, J. S. *et al*. Responses of grassland production to single and multiple global environmental changes. *PloS Bio.* **3**, 1829-1837 (2005).
7. Suttle, K. B., Thomsen, M. A. & Power, M. E. Species interactions reverse grassland responses to changing climate. *Science* **315,** 640-642 (2007).
8. Harpole, W. S., Potts, D. L., Suding, K. N. Ecosystem responses to water and nitrogen amendment in a California grassland. *Glob. Chang. Biol*. **13**, 2341-2348 (2007).
9. Miranda, J. d. D., Armas, C., Padilla, F. M. & Pugnaire, F. I. Climatic change and rainfall patterns: Effects on semi-arid plant communities of the Iberian Southeast *J. Arid Env.* **75**, 1302-1309 (2011).
10. Miranda J. D., Padilla, F. M., Lazaro, R., Pugnaire, F. I. Do changes in rainfall patterns affect semiarid annual plant communities? *J. Veg. Sci.* **20**, 269-276 (2009).
11. Prieto, P., Peñuelas J., Lloret, F., Llorens, L. & Estiarte, M. Experimental drought and warming decrease diversity and slow down post-fire succession in a Mediterranean shrubland. *Ecography* **32**, 623-636 (2009).
12. Lloret, F., Peñuelas, J., Prieto, P., Llorens, L., Estiarte, M. Plant community changes induced by experimental climate change: Seedling and adult species composition. *Persp. Plant. Ecol. Evol. Syst.* **11**, 53-63 (2009).

13. Peñuelas, J., *et al*. Response of plant species richness and primary productivity in shrublands along north-south gradient in Europe to seven years of experimental warming and drought: reductions in primary productivity in the heat and drought year of 2003. *Glob. Change Biol.* **13**, 2563-2581 (2007).
14. Evans, S. E., Byrne, K. M., Lauenroth, W. K. & Burke, I. C. Defining the limit to resistance in a drought-tolerant grassland: long-term severe drought significantly reduces the dominant species and increases ruderals. *J. Ecol*. **99,** 1500-1507 (2011).
15. Grime, P. J. *et al*. The response of two contrasting limestone grasslands to simulated climate change. *Science* **289**, 762-765 (2000).
16. Grime, P. J. *et al.* Long-term resistance to simulated climate change in an infertile grassland. *Proc. Natl. Acad. Sci. USA* **105**, 10028-10032 (2008).
17. Báez, S., Collins. S. L., Pockman, W. T., Johnson, J. E. & Small, E. E. Effects of experimental rainfall manipulation on Chihuahuan Desert grassland and shrubland plant communities. *Oecologia* **172**, 1117-1127 (2013).
18. Collins S. L. *et al.* Stability of tallgrass prairie during a 19-year increase in growing season precipitation. *Funct. Ecol*. **26**, 1450-1459 (2012).
19. Yang H. *et al*. Plant community responses to nitrogen addition and increased precipitation: the importance of water availability and species traits. *Glob. Chang. Biol.* **17**, 2936-2944 (2001).
20. Holzapfel, C., Tielbörger, K., Parag, H. A., Kigel, J.& Sternberg, M. Annual plant–shrub interactions along an aridity gradient. *Bas. Appl. Ecol.* **7,** 268-279 (2006).
21. Golodets, C. *et al.* From desert to Mediterranean rangelands: will increasing drought and inter-annual rainfall variability affect herbaceous annual primary productivity? *Clim. Chang.* **119**, 785-798 (2013).
22. Shafran-Nathan, R., Svoray, T. & Perevolotsky, A. The resilience of annual vegetation primary production subjected to different climate change scenarios. *Clim. Chang*. **118**, 227-243 (2013).
23. Smiatek, G., Kunstmann, H. & Heckl, A. High resolution climate change simulations for the Jordan River area. *J. Geophys. Res.* **116**, D16111, doi:10.1029/2010JD015313, (2011).

24. Samuels, R., Harel, M. & Alpert, P. A new methodology for weighting high-resolution model simulations to project future rainfall in the Middle East. *Clim Res*. **57**, 51-60 (2013).
25. Hemming, D., Buontempo, C., Burke, E., Collins, M. & Kaye, N. How uncertain are climate model projections of water availability indicators across the Middle East? *Phil. Trans. Royal. Soc. A* **368**, 5117–5135 (2010).
26. Yahdjian, L. & Sala, O. E. A rainout shelter design for intercepting different amounts of rainfall. *Oecologia* **133**, 95-101 (2002).
27. Hoenig, J. M., Heisey, D. M. The abuse of power: The pervasive fallacy of power calculations for data analysis. *Am. Stat*. **55**, 19-24 (2001).
28. Tukey, J. W. Tightening the clinical trial. *Control. Clin. Trials* **14**, 266-285 (1993).
